# Serum TNF-α as an indicator reflecting the severity of intestinal barrier injury in a neonatal rat model of necrotizing enterocolitis

**DOI:** 10.3389/fped.2026.1881600

**Published:** 2026-07-22

**Authors:** Li Zhang, Qi Jiang, Xin Liu, Xiaolin Zhao, Baiping Sun, Yubo Wang

**Affiliations:** 1 Department of Neonatology, Northwest Women’s and Children’s Hospital, Xi’an, China; 2 Graduate School, Shaanxi University of Chinese Medicine, Xianyang, China; 3 Department of Pediatric Surgery, Northwest Women’s and Children’s Hospital, Xi’an, China; 4 School of Life Science and Technology, Xidian University, Xi’an, China

**Keywords:** inflammatory cytokines, intestinal barrier, necrotizing enterocolitis, tight junction, TNF-α

## Abstract

**Background and objective:**

Necrotizing enterocolitis (NEC) is a severe gastrointestinal emergency in preterm infants and is characterized by progressive intestinal barrier injury accompanied by systemic inflammation. This study aimed to determine whether peripheral inflammatory signals reflect structural damage to the intestinal barrier in a graded neonatal rat model of NEC and to identify candidate blood-based indicators of disease severity.

**Methods:**

Neonatal Sprague-Dawley rats were assigned to Control, NEC, and NEC + LPS groups. Ileal injury was assessed by histopathology. The localization and expression of the tight junction proteins ZO-1 and Claudin-3 were evaluated by immunohistochemistry, Western blotting, and qPCR. TNF-α and IL-6 levels in ileal tissue and peripheral serum were measured by ELISA. Correlation analysis and leave-one-out cross-validation (LOOCV)-based regression models were used to assess the relationship between peripheral inflammatory markers and tight junction injury.

**Results:**

The model showed a clear severity gradient across groups. Histological injury progressively worsened from the NEC group to the NEC + LPS group. In parallel, ZO-1 and Claudin-3 showed progressively disrupted membrane localization and reduced protein and mRNA expression. TNF-α and IL-6 levels in both ileal tissue and serum increased with disease severity. Serum TNF-α and IL-6 were inversely associated with ileal ZO-1 and Claudin-3 expression in complete-case analyses. In exploratory regression analysis, the TNF-α-only model showed higher internal LOOCV performance than the IL-6-only model for estimating both tight junction proteins.

**Conclusion:**

NEC progression in neonatal rats was associated with progressive structural disruption of the intestinal barrier and increasing inflammatory activation. Within this experimental framework, serum TNF-α was more closely associated with tight junction injury than IL-6. These findings are preliminary and hypothesis-generating, and serum TNF-α should not yet be considered a validated clinical biomarker before confirmation in larger animal studies, time-course experiments, and clinical cohorts.

## 1 Introduction

Necrotizing enterocolitis (NEC) is a severe gastrointestinal emergency of the neonatal period, particularly in preterm infants (1). and may progress rapidly from non-specific abdominal symptoms to intestinal necrosis, perforation, septic shock, and multi-organ failure (2, 3). Current severity assessment relies mainly on Bell staging, radiographic findings, and repeated clinical observation; however, these approaches often become clearly informative only after structural intestinal damage has developed (4, 5).Therefore, experimentally grounded indicators that reflect intestinal injury severity may help guide future biomarker development and disease monitoring.

Peripheral blood biomarkers are attractive because they can be measured repeatedly and less invasively than tissue-based markers (6). C-reactive protein (CRP), procalcitonin (PCT), and routine blood indices are commonly used to reflect systemic inflammation, but they have limited specificity in neonates and may overlap with sepsis or other inflammatory conditions (7, 8), Cytokines may be more directly related to disease-associated inflammatory pathways, yet their clinical interpretation is affected by biological timing, assay platform variability, and sample-handling factors31. TNF-α is closely involved in upstream inflammatory signaling and barrier-related injury in NEC (9, 10). whereas IL-6 is more commonly interpreted as part of the downstream systemic acute-phase response (11, 12),. This difference in biological hierarchy should be considered when comparing these two markers.

The central pathological feature of NEC is disruption of intestinal barrier integrity (13). Tight junctions (TJs) between intestinal epithelial cells are essential for barrier function and are maintained by transmembrane proteins such as Claudin-3 and Occludin, together with scaffold proteins such as ZO-1 (14). During NEC progression, tight junction proteins may show reduced expression and abnormal membrane localization, reflecting structural barrier injury (15).For example, decreased Claudin-3 expression has been reported to help differentiate NEC from focal intestinal perforation, and the expression levels of various junction-related proteins have been associated with clinical outcomes (16) Because direct assessment of these tissue markers is difficult in clinical practice, it remains important to determine whether circulating inflammatory signals are associated with tissue-defined tight junction injury.

Tight-junction proteins can directly characterize structural barrier injury, but their tissue localization makes repeated assessment difficult in clinical settings. In contrast, peripheral blood inflammatory cytokines can be measured more readily and dynamically, yet whether they correspond to tissue-defined barrier injury remains unclear (17). To address this question, the present study established a graded neonatal rat NEC model and performed an exploratory analysis of the quantitative associations between serum TNF-α/IL-6 levels and intestinal ZO-1/Claudin-3 expression across Control, NEC, and NEC + LPS groups. A disease-only analysis was also conducted to evaluate whether these associations were maintained within diseased animals. Regression models were used descriptively to estimate tight junction protein expression from peripheral inflammatory factors, and LOOCV was applied as an internal exploratory assessment rather than definitive validation. The study was designed to generate experimental evidence for candidate peripheral indicators of NEC-associated intestinal barrier injury, not to establish a clinically validated biomarker.

## 2 Methods

### 2.1 Animal ethics and NEC model establishment

A graded neonatal rat model was established to evaluate intestinal injury, inflammatory responses, and tight junction disruption across three experimental conditions. Neonatal Sprague-Dawley (SD) rats within 24 h of birth were used in this study. All animals were supplied by Vital River Laboratory Animal Technology Co., Ltd. [Beijing, China; production license No. SCXK (Chuan) 2025-0030] and then randomly divided into three groups (*n* = 12): Control group, NEC group, and NEC + LPS group. All animals were housed under standard environmental conditions (22 ± 2 °C, 12 h light/dark cycle). The experimental protocol was approved by the Animal Ethics Committee of Xi'an Jiaotong University (XJTUAE2025-3562) and complied with the Guidelines for the Care and Management of Laboratory Animals.

The NEC model was established based on a classic neonatal rat NEC modeling method with modifications (18). Pups in the Control group were naturally nursed by their dams. In the NEC group, pups were subjected to artificial feeding with hyperosmolar formula milk combined with hypoxia and cold exposure for 96 consecutive hours. The formula milk consisted of 15 g casein, 5 g soybean oil, and 10 g glucose, diluted with distilled water to a final volume of 100 mL, sterilized at 121 °C, and stored at 4 °C. Artificial feeding was administered five times daily. During the modeling period, pups were subjected to hypoxia (95% N₂/5% O₂, 10 min/session) twice daily, followed by cold exposure at 4 °C (10 min/session). In the NEC + LPS group, in addition to the NEC modeling protocol, LPS was administered simultaneously with formula milk via oral gavage daily at 8:00 AM (2 mg/kg) to enhance inflammatory stimulation and exacerbate intestinal mucosal injury.

Clinical sickness scores were assessed for all animals at the end of the modeling period using a standardized scoring system (19, 29). The scoring criteria encompassed four parameters: appearance, natural activity, response to touch, and body colour, with each parameter graded on a scale of 0 to 3, yielding a total score ranging from 0 to 12, as shown in Table 1. Higher scores indicated more severe clinical illness.

**Table 1:** Clinical sickness score for assessment of neonatal rat clinical status (29).

Score	Appearance	Natural activity	Response to touch
0	Tonic and well-hydrated	Moving normally	Alert
1	Slimmer, but still tonic and hydrated	Able to wriggle if put supine	Responding to mild stimulation
2	Skinny, floppy and dehydrated	Not able to wriggle if put supine	Responding to vigorous stimulation
3	Gasping and in agony	Not moving limbs and lying still	Unresponsive

### 2.2 Sample collection and processing

At the end of the modeling period, all neonatal rats were anesthetized by intraperitoneal injection of sodium pentobarbital (50 mg/kg). Whole blood was collected by cardiac puncture. Blood samples were allowed to stand at room temperature for 2 h and were then centrifuged at 3,000 × g for 15 min at 4 °C to separate the serum. The serum was aliquoted and stored at −80 °C for subsequent analysis.

Ileal tissue approximately 3 cm proximal to the ileocecal junction was rapidly collected. One portion was fixed in 4% paraformaldehyde for histopathological and immunohistochemical analysis; the remaining tissue was snap-frozen in liquid nitrogen and transferred to −80 °C for subsequent protein and RNA extraction.

### 2.3 Histopathological evaluation

Ileal tissue samples were paraffin-embedded, sectioned at 4 μm, and stained with hematoxylin and eosin (H&E). Intestinal injury was independently evaluated by two investigators blinded to group allocation using previously established histopathological criteria (19). The histological grading system was defined as follows: 0: Normal intact villous structure; 1, Mild injury with widening of intervillous spaces; 2, Moderate injury with epithelial shedding at villous tips; 3, Severe injury with extensive villous epithelial shedding; and 4, Necrosis with disintegration of the lamina propria and/or perforation. The mean score from the two investigators was used for subsequent statistical analysis.

### 2.4 Inflammatory cytokine measurement

Levels of tumor necrosis factor-alpha (TNF-α) and interleukin-6 (IL-6) in serum and ileal tissue homogenates were measured using enzyme-linked immunosorbent assay (ELISA) kits（TNF-α: BioRuler, China, Cat. No. RA20035, 48 T; IL-6: BioRuler, China, Cat. No. RA20607, 48T） according to the manufacturers’ instructions. Each sample was assayed in duplicate, and the mean values were used for analysis.

### 2.5 Analysis of tight junction protein expression

#### 2.5.1 Immunohistochemistry (IHC)

Paraffin-embedded sections were deparaffinized, rehydrated, and subjected to antigen retrieval. Sections were then incubated with primary antibodies against Claudin-3 (Bioss, China; Cat. No. bs-2788R; 50 μl)(1:200 dilution) and ZO-1 (Bioss, China; Cat. No. bs-0155R; 50 μl) (1:150 dilution), followed by visualization with a DAB detection kit. The localization and staining intensity of Claudin-3 and ZO-1 in the epithelial cell membranes of ileal villi were examined under a light microscope.

#### 2.5.2 Western blot

Total protein was extracted from ileal tissue samples and quantified. Equal amounts of protein were separated by electrophoresis, transferred onto membranes, and incubated with primary antibodies against Claudin-3, ZO-1, and GAPDH as an internal control. Protein bands were visualized by chemiluminescence, and band intensities were analyzed using ImageJ software. Semi-quantitative analysis was performed by calculating the ratio of the target protein band intensity to that of GAPDH.

#### 2.5.3 Real-Time quantitative PCR (qPCR)

Total RNA was extracted from ileal tissue using TRIzol reagent and reverse transcribed into cDNA. Amplification was performed using the SYBR Green method. The relative mRNA expression levels of Claudin-3 and ZO-1 were calculated using the 2^−*ΔΔ*Ct method, with *β*-actin serving as the internal reference gene.

### 2.6 Supportive biological assessments

#### 2.6.1 Apoptosis assay

Single-cell suspensions were prepared from fresh ileal tissue samples. Apoptosis was assessed using an APC-Annexin V/PE-PI apoptosis detection kit according to the manufacturer's instructions. Briefly, cells were harvested, washed with PBS, and incubated with APC-Annexin V and PE-PI staining solution in the dark at room temperature. Unstained controls were included for determining the negative boundary and quadrant setting, and two single-stained controls (samples stained with APC-Annexin V alone and samples stained with PE-PI alone) were included for fluorescence compensation adjustment. Flow cytometric analysis was performed using a FongCY C2080 flow cytometer, and data were analyzed with FlowJo v10.8.1. A total of 20,000 events were acquired for each sample. Early apoptotic cells were defined as Annexin V^+^/PI^−^, and late apoptotic cells were defined as Annexin V^+^/P ^+^ . The total apoptosis rate was calculated as the sum of early and late apoptotic cells.

#### 2.6.2 Routine blood analysis

Whole blood collected via cardiac puncture was immediately transferred to tubes containing EDTA-K2 anticoagulant and gently inverted for mixing. Samples were analyzed using an automated animal hematology analyzer. The measured parameters included: white blood cell count (WBC), absolute neutrophil count (NEU), lymphocyte percentage (LYM%), hemoglobin concentration (HGB), hematocrit (HCT), and large platelet count (P-LCC). These analyses were performed to assess the systemic inflammatory burden and volume status in the model animals.

### 2.7 Statistical analysis

Data were expressed as mean ± standard deviation (x¯ ± s). Standard group-level statistical analyses were performed using SPSS software, version 26.0. Comparisons among multiple groups were performed using one-way analysis of variance (ANOVA), followed by the least significant difference (LSD)-t test for pairwise comparisons. Complete-case correlation analysis, bootstrap confidence interval estimation, exploratory regression modeling, baseline model comparison, and permutation testing were performed using MATLAB R2025a.

#### 2.7.1 Sample inclusion for paired analyses

The original animal experiment included 12 animals per group. Group-level analyses used all available animals with corresponding measurements. Correlation and regression analyses required complete paired measurements from the same animal, including serum TNF-α, serum IL-6, ileal ZO-1 protein expression, and ileal Claudin-3 protein expression. Therefore, these analyses used a complete-case dataset, and no data imputation was performed.

The pooled complete-case dataset included nine animals, with three animals from each group. This dataset was used for the pooled correlation analysis and all regression, baseline-model, and permutation analyses. A disease-only complete-case subset including the NEC and NEC + LPS groups (*n* = 6) was used only for the secondary disease-only correlation analysis.

#### 2.7.2 Correlation analysis

Spearman's rank correlation analysis was used to examine the associations between serum TNF-α and IL-6 levels and ileal ZO-1 and Claudin-3 protein expression. The pooled analysis included the Control, NEC, and NEC + LPS groups and was used to assess whether serum inflammatory markers tracked the overall experimental severity gradient. The disease-only analysis included only the NEC and NEC + LPS groups and was used as a secondary analysis to examine whether the inverse associations were also observed within diseased animals.

Spearman correlation coefficients (*ρ*) and two-sided *p*-values were calculated for each marker pair. The 95% confidence intervals were estimated using nonparametric bootstrap resampling with 5000 iterations. For the pooled analysis, the four *p*-values were adjusted using the Benjamini-Hochberg false discovery rate method. The forest plot shows the correlation coefficients and bootstrap 95% confidence intervals for both analyses, with FDR-adjusted q-values reported for the pooled analysis.

#### 2.7.3 Exploratory regression analysis

Linear regression models were used to explore whether serum inflammatory markers could estimate ileal tight junction protein expression in the pooled complete-case dataset. Log10-transformed ileal Claudin-3 and ZO-1 protein expression levels were used as dependent variables. Three cytokine-based models were evaluated for each outcome, including TNF-α only, IL-6 only, and TNF-α plus IL-6. A mean-only baseline model was also evaluated by predicting each held-out value from the mean outcome value of the training samples.

Model performance was assessed using leave-one-out cross-validation (LOOCV). In each iteration, one sample was held out, and the model was trained using the remaining eight samples. For cytokine-based models, predictors were standardized within each training fold, and the held-out sample was transformed using the corresponding training-fold mean and standard deviation.

Cross-validated coefficient of determination (R^2^cv), root mean square error (RMSE), and mean absolute error (MAE) were calculated from the observed and LOOCV-predicted values. R^2^cv was calculated as 1 minus the ratio between the LOOCV residual sum of squares and the total sum of squares around the observed mean. Negative R^2^cv values were retained.

Permutation testing was performed to assess whether the observed LOOCV performance exceeded chance-level performance. For each cytokine-based model, the predictor values were kept unchanged, whereas the outcome values were randomly permuted across samples. The complete LOOCV procedure was repeated for 5,000 permutations. The empirical permutation *p*-value was defined as the fraction of permutation results that reached or exceeded the observed R^2^cv value. A standard one-count correction was applied to avoid zero p-values. Because of the small complete-case sample size, all LOOCV results were interpreted as exploratory internal performance estimates rather than externally validated predictive performance.

## 3 Results

### 3.1 Establishment of the NEC model

**Figure 1:**
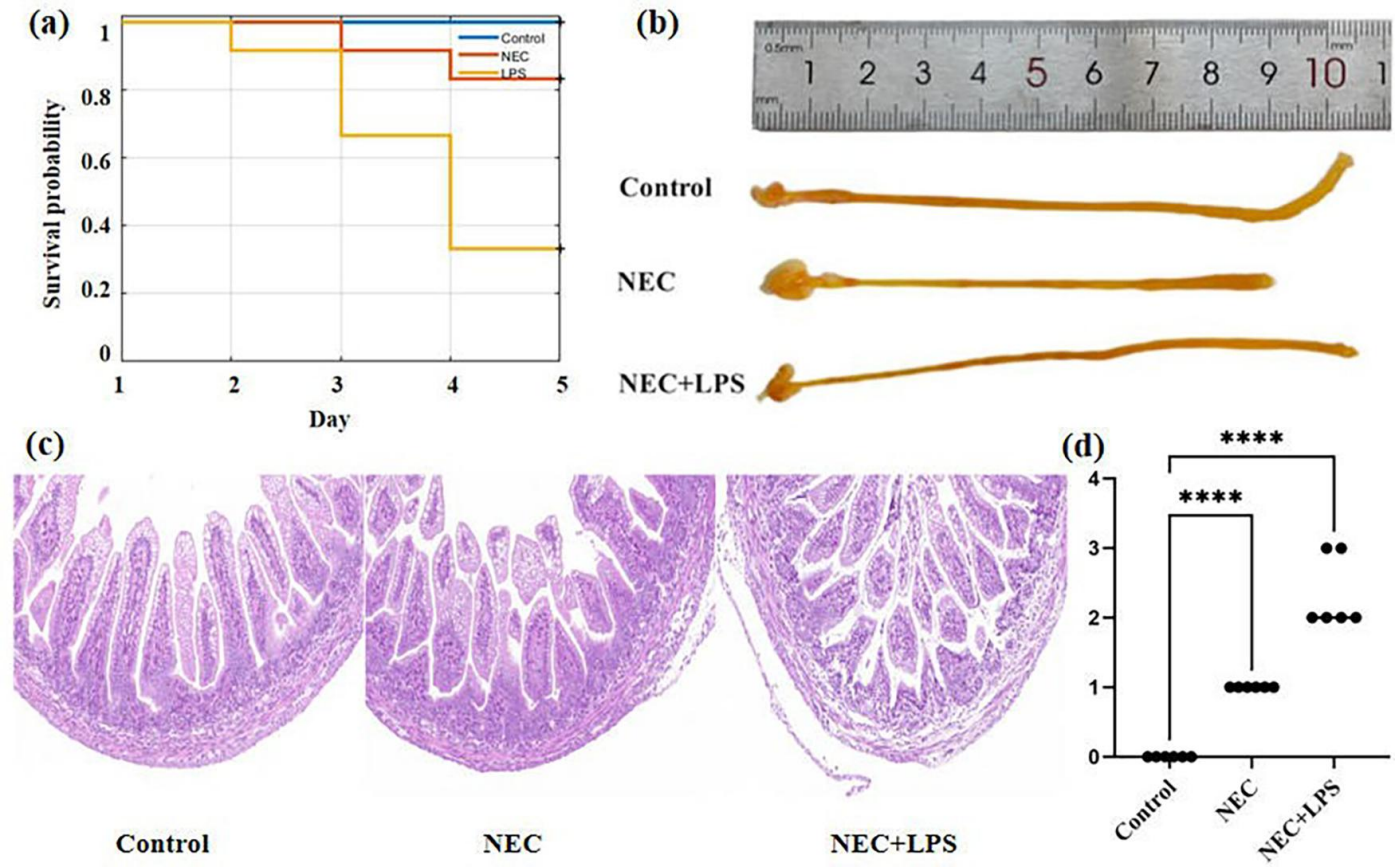
Establishment of the graded NEC rat model. **(a)** Kaplan–Meier survival analysis showing reduced survival in the NEC and NEC + LPS groups compared with the Control group. **(b)** Representative gross morphology of intestinal segments showing progressive shortening and congestion in the NEC and NEC + LPS groups. **(c)** Representative H&E-stained ileal sections showing intact villous architecture in the Control group, moderate villous disruption in the NEC group, and severe mucosal injury with marked villous destruction in the NEC + LPS group.**(d)** Histopathological injury scores showing progressively increased ileal damage across groups.

Kaplan–Meier survival analysis revealed a significantly reduced survival rate in the NEC group and the NEC + LPS group compared to the control group (log-rank test, *χ*^2^ = 494.888, df = 2, *p* < 0.001), with the NEC + LPS group exhibiting the highest mortality, as shown in Figure 1a. Gross examination of the intestinal segments revealed progressive shortening and congestion from the NEC group to the NEC + LPS group, whereas the Control group showed normal morphology, as shown in Figure 1b. Histologically, H&E staining of the ileum showed intact villous architecture, continuous epithelium, and orderly crypts in the Control group. In contrast, the NEC group showed disrupted villous architecture, epithelial shedding, and lamina propria injury. These changes were more severe in the NEC + LPS group, which displayed extensive mucosal destruction, villous loss, and marked inflammatory cell infiltration, as shown in Figure 1d. Blinded pathological scoring further confirmed this graded pattern of injury, with significantly higher scores in the NEC and NEC + LPS groups than in the Control group, and the highest scores observed in the NEC + LPS group, as shown in Figure 1c.

Assessment of physiological or clinical manifestation scores also showed significant differences among groups, as reported in Table 2. Compared with the Control group, scores were significantly higher in both the NEC and NEC + LPS groups, and the NEC + LPS group also showed significantly higher scores than the NEC group. These findings collectively indicate that the model reproduced a graded disease pattern across the three groups.

**Table 2:** Animal clinical manifestation score.

Comparison	Mean Difference (MD)	Standard Error (SE)	t	P
Control vs NEC	−1.33	0.2	−6.65	<0.001
Control vs NEC + LPS	−2.25	0.2	−11.25	<0.001
NEC vs NEC + LPS	−0.92	0.2	−4.6	<0.001

### 3.2 Peripheral blood parameters reflect systemic inflammatory response and hemoconcentration

**Figure 2:**
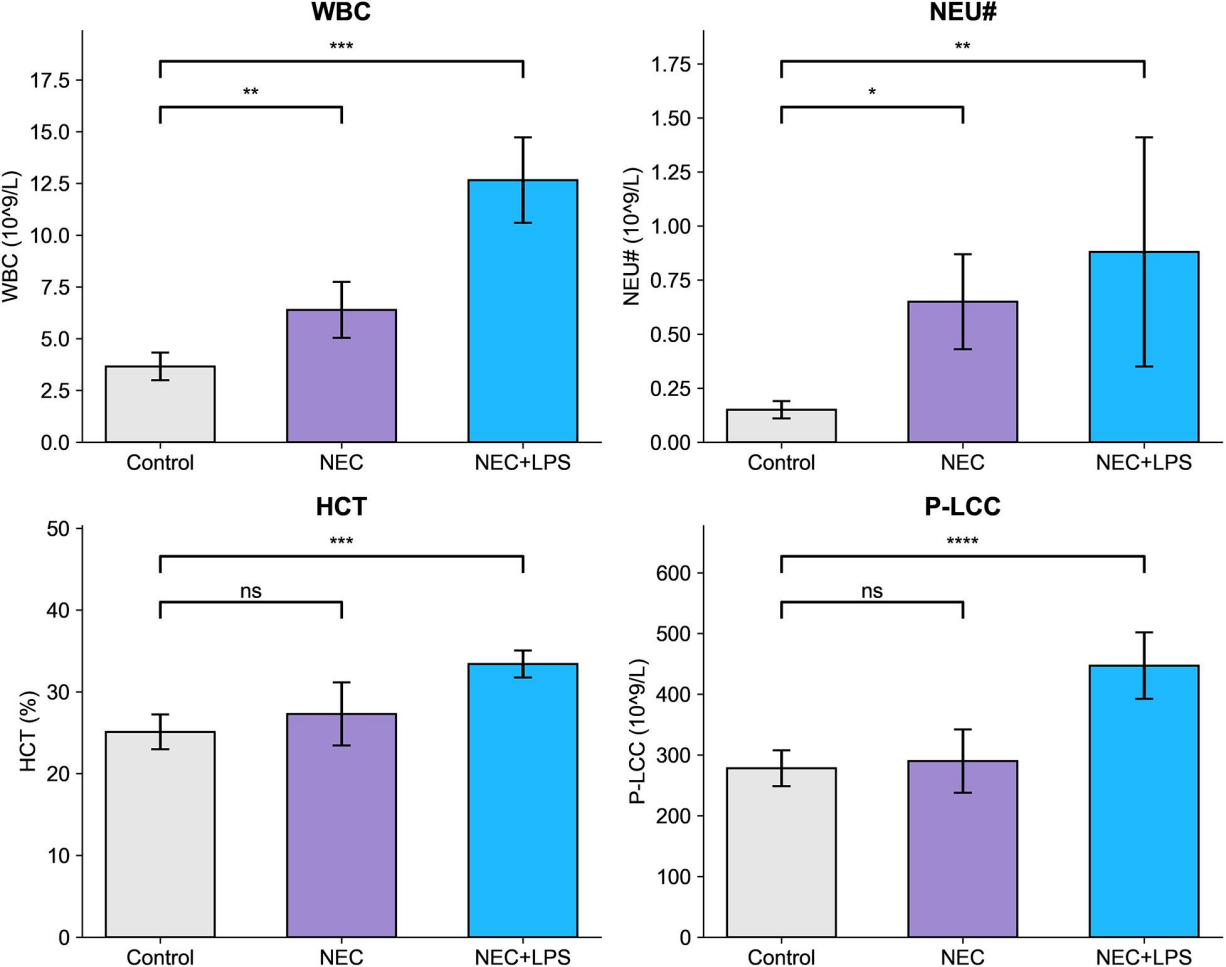
Peripheral blood parameters reflect systemic inflammatory response and hemoconcentration.

Analysis of peripheral blood parameters revealed progressive alterations with increasing disease severity, the results are summarized in Table 3 and Figure 2. Total white blood cell (WBC) counts were significantly increased in the NEC group and were further elevated in the NEC + LPS group. and neutrophil (NEU) counts increased significantly with disease severity. The NEC + LPS group showed WBC counts 12.65 ± 2.05 × 10⁹/L, compared with 3.66 ± 0.67 × 10⁹/L in the control group (*P* < 0.001). Neutrophil (NEU) counts showed a similar trend, with marked elevation in both disease groups and the highest levels in the NEC + LPS group.

**Table 3:** Comparison of key CBC.

Parameter	Unit	Control	NEC	NEC + LPS
WBC	10⁹/L	3.66 ± 0.67	6.39 ± 1.28**	12.66 ± 1.96***
NEU#	10⁹/L	0.15 ± 0.04	0.70 ± 0.23*	0.89 ± 0.52**
LYM	%	93.42 ± 1.22	84.18 ± 5.37	89.20 ± 5.75
HGB	g/L	86.83 ± 7.08	97.83 ± 11.53	111.33 ± 7.23**
HCT	%	25.07 ± 2.22	27.28 ± 3.95	33.40 ± 1.54***
P-LCC	10⁹/L	278.17 ± 28.52	289.83 ± 52.12	447.00 ± 53.67***

Compared with Control group.

* *P* < 0.05.

** *P* < 0.01.

*** *P* < 0.001.

Hemoglobin (HGB) and hematocrit (HCT) also increased with disease severity. Among these parameters, HCT showed a clear stepwise rise, reaching 33.43 ± 1.66% in the NEC + LPS group compared with 25.10 ± 2.13% in the control group (*P* < 0.001). In addition, platelet large cell count (P-LCC) was markedly elevated in the NEC + LPS group. In contrast, lymphocyte percentage (LYM%) showed no statistically significant change across groups.

Overall, from the Control group to the NEC group and further to the NEC + LPS group, systemic inflammatory activation and hemoconcentration progressively intensified. WBC count increased from 3.66 × 10⁹/L to 6.39 × 10⁹/L (NEC group, *P* < 0.01) and further to 12.66 × 10⁹/L (NEC + LPS group, *P* < 0.001), representing a total increase of 246%. HCT increased from 25.07% to 27.28% and further to 33.40% (*P* < 0.001), representing a total increase of 33.2%. Thus, the relative increase in WBC was more prominent than the increase in HCT, suggesting that systemic inflammatory activation was a major feature of the graded NEC model rather than being explained only by hemoconcentration.

### 3.3 Intestinal barrier tight junction proteins Are progressively disrupted in NEC

**Figure 3:**
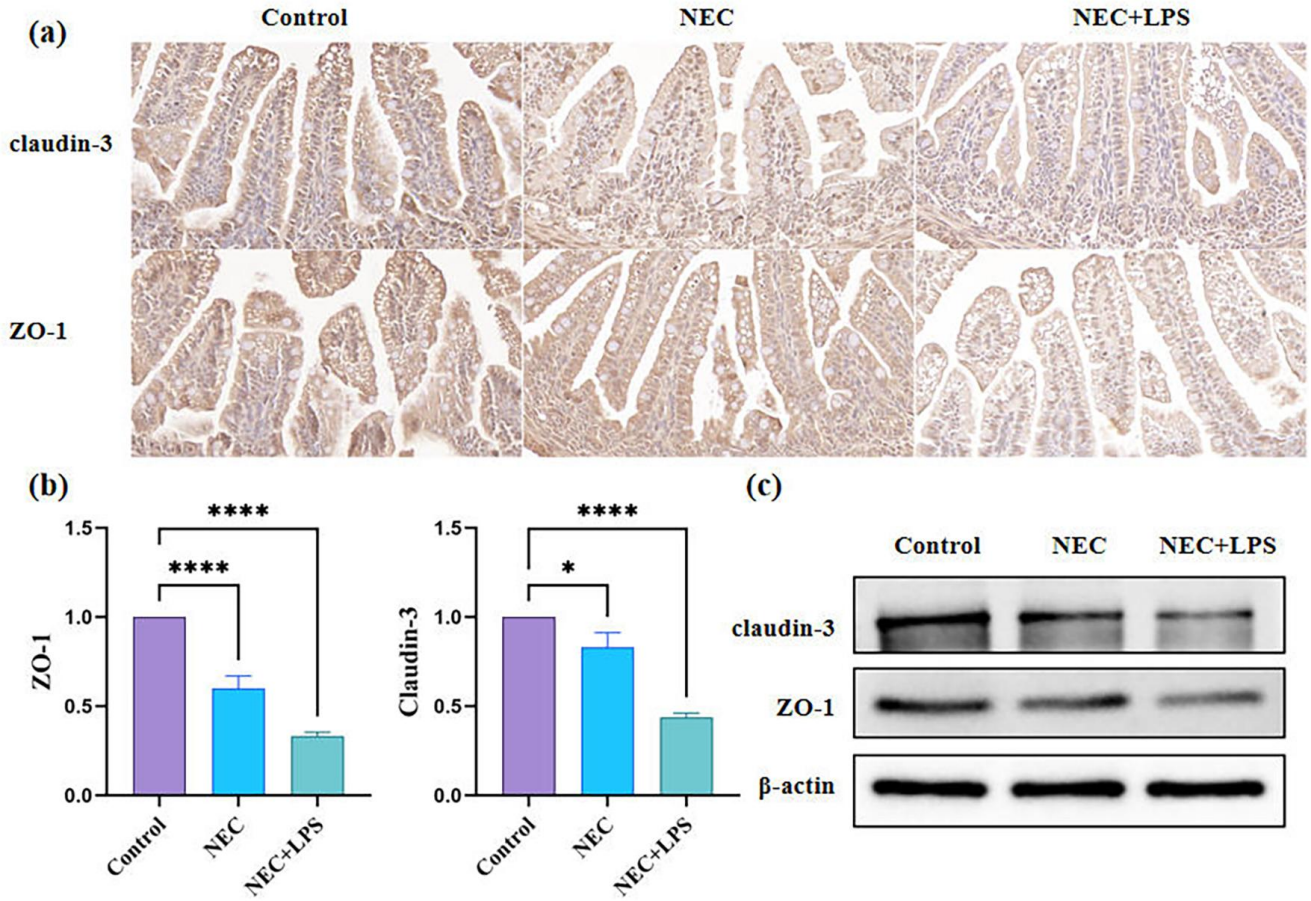
Progressive disruption of intestinal tight junction proteins in NEC. **(a)** Immunohistochemical staining showed that both Claudin-3 and ZO-1 exhibited continuous membrane localization in the Control group, and progressively disrupted staining in the NEC and NEC + LPS groups. **(b)** qPCR analysis of ZO-1 and Claudin-3 mRNA expression. **(c)** Western blot analysis of ZO-1 and Claudin-3 protein expression in ileal tissue.

Immunohistochemical analysis showed that ZO-1 and Claudin-3 were continuously distributed along the epithelial cell membranes of ileal villi in the Control group. In the NEC group, both proteins showed discontinuous membrane localization, reduced staining intensity, and focal loss. These alterations were more pronounced in the NEC + LPS group, in which membrane staining was further disrupted and structural integrity was extensively lost, as shown in Figures 3a,b.

Consistent with the immunohistochemical findings, Western blot analysis showed that protein expression levels of ZO-1 and Claudin-3 were reduced in both the NEC and NEC + LPS groups compared with the Control group, with the lowest expression levels observed in the NEC + LPS group (Figure 3c, *p* < 0.05). qPCR analysis further showed that the relative mRNA expression levels of both ZO-1 and Claudin-3 were reduced in the NEC and NEC + LPS groups, consistent with the protein-level changes, as shown in Figures 3d,e. Together, these findings indicate progressive disruption of intestinal tight junction structure and expression across the three groups.

### 3.4 Inflammatory cytokines in ileal tissue and Serum increase with disease severity

**Figure 4:**
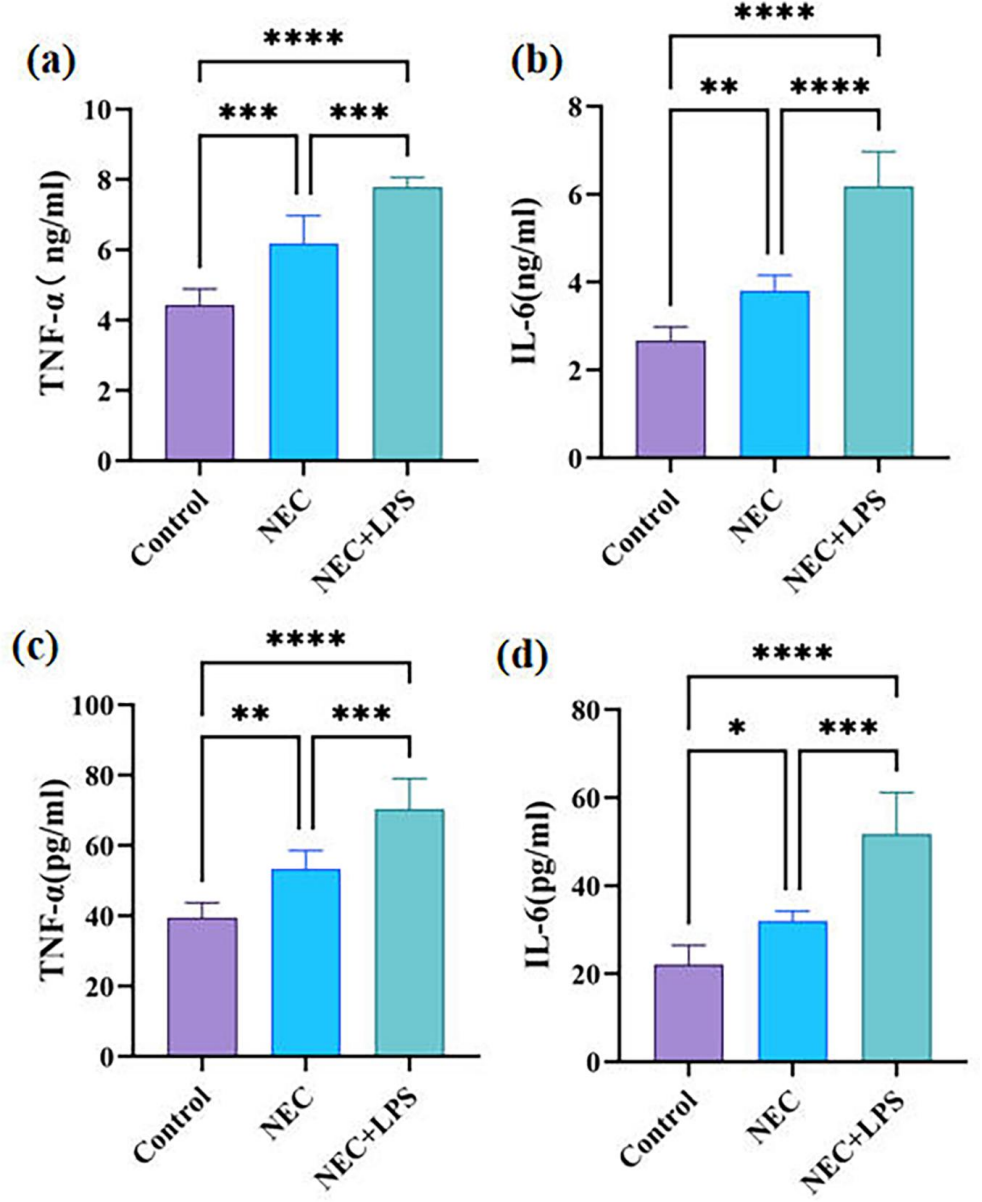
TNF-α and IL-6 levels in ileal tissue and serum. **(A,B)** ELISA analysis of TNF-α and IL-6 in ileal tissue homogenates. **(C,D)** ELISA analysis of TNF-α and IL-6 in peripheral serum. Both tissue and serum cytokine levels increased progressively from the Control group to the NEC group and were highest in the NEC + LPS group. *P < 0.05, **P < 0.01, ***P < 0.001, ****P < 0.0001.

ELISA analysis showed that TNF-α and IL-6 levels were significantly elevated in both ileal tissue homogenates and peripheral serum in the NEC and NEC + LPS groups compared with the Control group, as shown in Figure 4. In both compartments, cytokine levels were lowest in the Control group, increased in the NEC group, and reached the highest levels in the NEC + LPS group. This consistent pattern was aligned with the severity gradient of intestinal injury.

### 3.5 Apoptosis of intestinal epithelial cells increases with barrier injury severity

**Figure 5:**
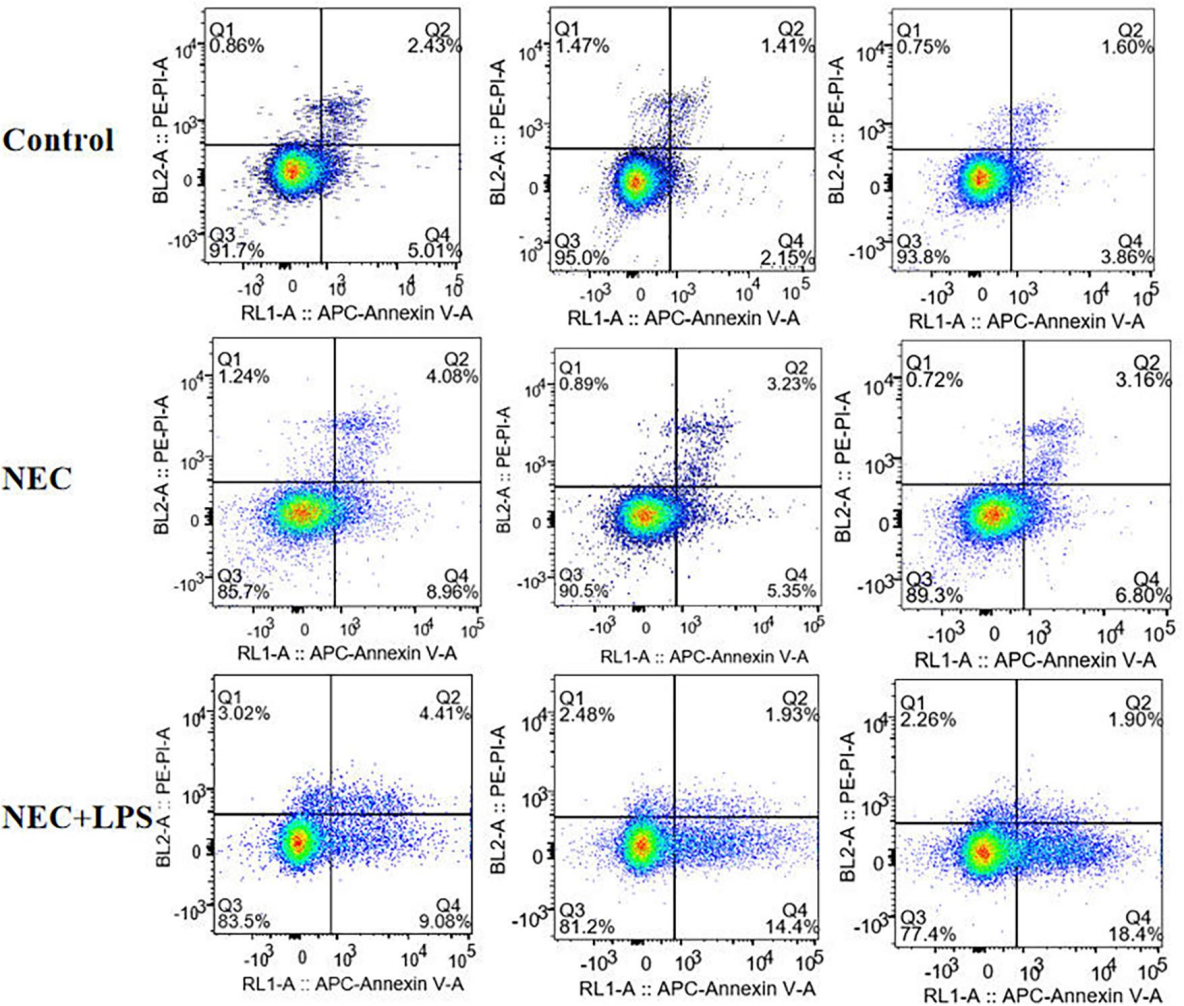
APC-Annexin V/PE-PI flow cytometric analysis of ileal epithelial cell apoptosis.**(a)** Representative Annexin V/PI flow cytometry plots for the Control, NEC, and NEC + LPS groups. **(b)** Quantification of early apoptotic, late apoptotic, and total apoptotic cells. The total apoptotic rate increased progressively across groups, with early apoptosis contributing substantially to the increase observed in the NEC and NEC + LPS groups.*P < 0.05, **P < 0.01, ***P < 0.001, ****P < 0.0001.

Flow cytometric analysis using APC-Annexin V/PE-PI double staining demonstrated a progressive increase in apoptosis in ileal tissue-derived cells with increasing disease severity, as shown in Figure 5. The total apoptosis rate in the Control group was 5.49 ± 1.94%. This rate increased to 10.53 ± 2.28% in the NEC group and further increased to 16.71 ± 3.42% in the NEC + LPS group. The increase in total apoptosis was primarily associated with early apoptosis, which progressively increased from the Control group to the NEC group and reached its highest level in the NEC + LPS group. In contrast, late apoptosis showed more limited variation among groups. These findings were consistent with the graded pattern of intestinal barrier injury observed in the histological and tight junction analyses.

### 3.6 Serum inflammatory factors Are negatively associated with intestinal tight junction protein expression

**Figure 6:**
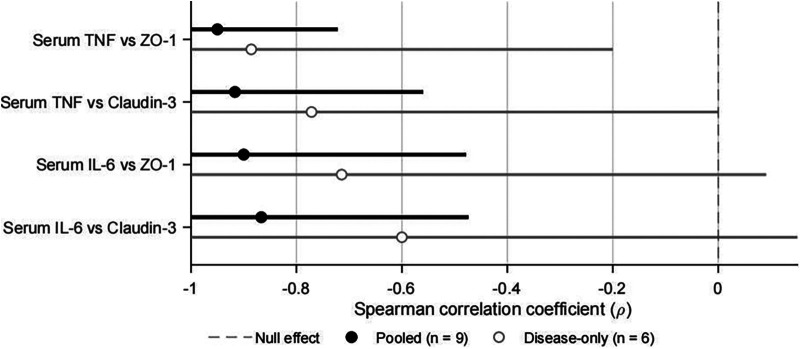
Correlations between serum inflammatory factors and intestinal tight junction protein expression in complete-case analyses. Spearman correlations were calculated in the pooled complete-case dataset (Control, NEC, and NEC + LPS, *n* = 9) and in the disease-only subset (NEC and NEC + LPS, *n* = 6). Horizontal lines indicate bootstrap 95% confidence intervals. FDR-adjusted q-values are reported for the pooled analysis.

To evaluate whether peripheral inflammatory factors were associated with intestinal barrier injury, Spearman correlation analysis was performed between serum TNF-α and IL-6 levels and intestinal ZO-1 and Claudin-3 protein expression. Two analyses were conducted: a pooled analysis including all groups (Control, NEC, and NEC + LPS; *n* = 9) and a disease-only analysis including the NEC and NEC + LPS groups (*n* = 6). The results were shown in Figure 6.

In the pooled analysis, serum TNF-α and IL-6 were both negatively correlated with intestinal ZO-1 and Claudin-3 expression. All four pooled associations remained significant after FDR correction, with the strongest inverse association observed between serum TNF-α and ZO-1 (*ρ*=−0.950, q = 0.00035). The other pooled associations were also strongly negative, including serum TNF-α vs. Claudin-3 (*ρ*=−0.917, q = 0.0010), serum IL-6 vs. ZO-1 (*ρ*=−0.900, q = 0.0013), and serum IL-6 vs. Claudin-3 (*ρ*=−0.867, q = 0.0025).

In the disease-only analysis, the point estimates remained negative for all four marker pairs, but the correlations were weaker overall and the confidence intervals were wider than those in the pooled analysis. This pattern suggests that the stronger pooled associations largely reflected the between-group severity gradient across the Control, NEC, and NEC + LPS groups, whereas the within-disease analysis provided less precise evidence for these associations.

### 3.7 Exploratory regression analysis shows closer endpoint association for TNF-α than IL-6

**Figure 7:**
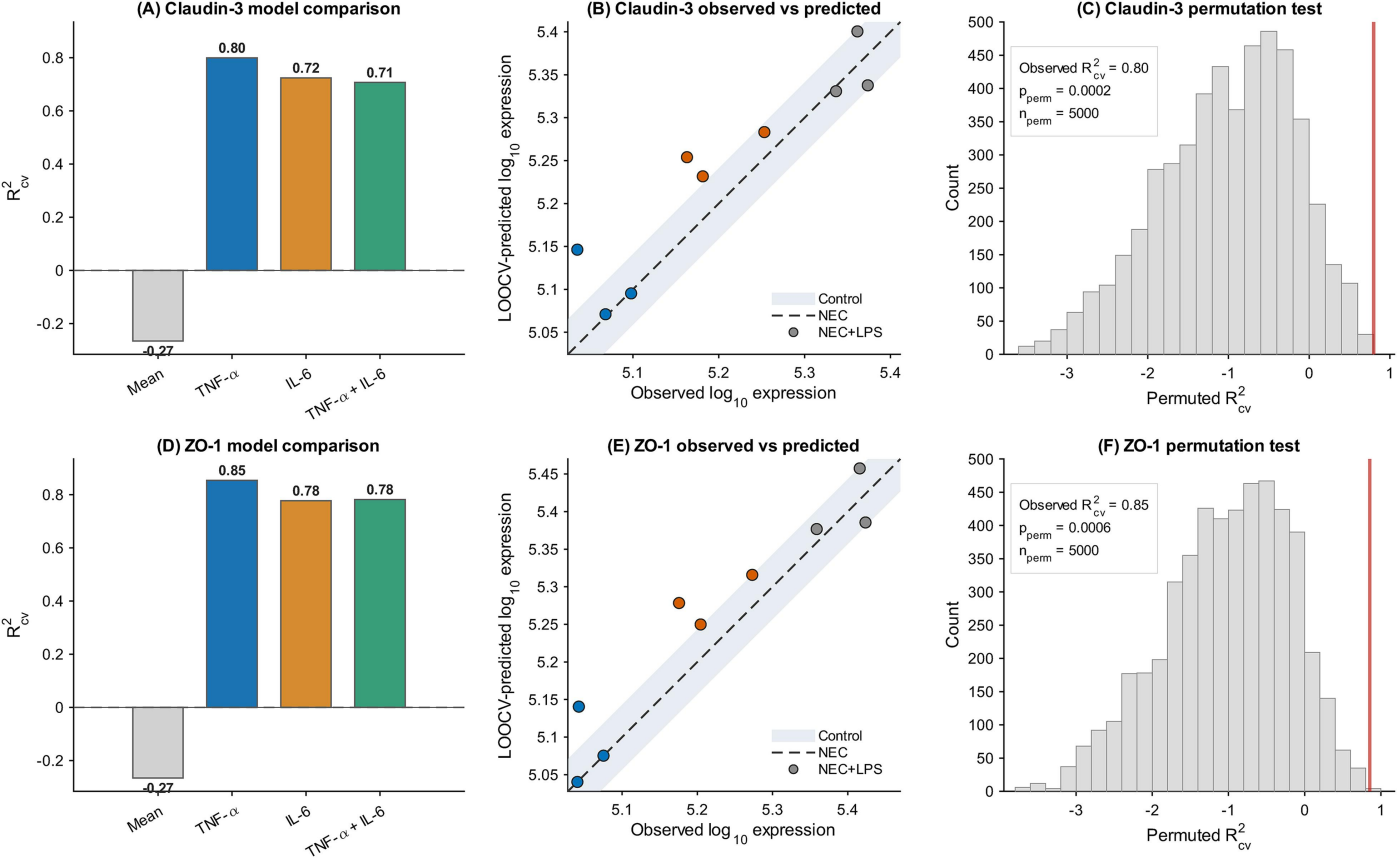
Exploratory LOOCV robustness analysis in the complete-case dataset. **(A,D)** Model comparison for log10-transformed Claudin-3 and ZO-1 expression. **(B,E)** Observed versus LOOCV-predicted values for the best cytokine-based model. The dashed line indicates the identity line, and the shaded band indicates the ± MAE range. **(C,F)** Permutation distributions of R^2^cv for the TNF-α-only models. The red line indicates the observed R^2^cv. Analyses used nine complete paired samples, with three animals per group.

To further examine whether serum inflammatory markers reflected intestinal tight junction injury, exploratory regression analysis was performed using the pooled complete-case dataset of nine animals. Log10-transformed Claudin-3 and ZO-1 protein expression levels were used as outcomes. Four models were compared for each outcome, including a mean-only baseline model, a TNF-α-only model, an IL-6-only model, and a combined TNF-α plus IL-6 model. The results are shown in Figure 7.

For Claudin-3, the TNF-α-only model showed the highest internal LOOCV performance, with an R^2^cv of 0.799, RMSE of 0.0547, and MAE of 0.0409. This performance was higher than that of the mean-only baseline model (R^2^cv = −0.266), the IL-6-only model (R^2^cv = 0.724), and the combined model (R^2^cv = 0.707). Permutation testing showed that the observed R^2^cv of the TNF-α-only model was higher than expected under random outcome permutation (pperm = 0.0002).

A similar pattern was observed for ZO-1. The TNF-α-only model again showed the highest internal LOOCV performance, with an R^2^cv of 0.854, RMSE of 0.0554, and MAE of 0.0430. This performance was higher than that of the mean-only baseline model (R^2^cv = −0.266), the IL-6-only model (R^2^cv = 0.777), and the combined model (R^2^cv = 0.782). Permutation testing also showed that the observed R^2^cv of the TNF-α-only model was higher than expected under random outcome permutation (pperm = 0.0006).

These results indicate that serum TNF-α showed a closer endpoint association with ileal Claudin-3 and ZO-1 expression than IL-6 in this complete-case dataset. However, because the analysis was based on only nine samples distributed across three experimental severity groups, the LOOCV results should be interpreted as exploratory internal estimates rather than externally validated predictive performance.

## 4 Discussion

The present study established a model with different severity levels of NEC that showed clear differences in survival, histopathological injury, and hematological alterations across groups, providing a useful framework for examining the relationship between systemic inflammation and intestinal barrier damage. Within this model, ZO-1 and Claudin-3 were evaluated as structural indicators of tight junction injury at three levels, including tissue localization, protein expression, and transcriptional expression. The findings from immunohistochemistry, Western blotting, and qPCR were consistent, showing that as disease severity increased, the distribution of ZO-1 and Claudin-3 changed from continuous membrane localization to discontinuity, fragmentation, or loss, accompanied by stepwise decreases in both protein and mRNA expression. These results support the presence of progressive tight junction injury during NEC progression.

From a mechanistic perspective, ZO-1 is a key scaffold protein in the tight junction complex that links transmembrane proteins to the actin cytoskeleton and helps maintain junctional stability (20). Claudin-3 is a representative sealing protein that directly contributes to the closure of paracellular pathways and the regulation of epithelial permeability (21). Our morphological and molecular observations during the progression of NEC align with the tight junction alterations reported in human NEC specimens by Kollmann et al. (22). and with findings from neonatal rat NEC models (23). Compared with previous studies reling on a single detection platform, the current study combined histological localization, protein quantification, and transcriptional analysis, allowing a more integrated characterization of the progression from abnormal junctional distribution to reduced expression during NEC pathogenesis.

A key exploratory observation of this study was that serum TNF-α showed a significant negative association with intestinal ZO-1 and Claudin-3 expression and yielded higher numerical internal LOOCV performance than IL-6 in the endpoint regression models. This pattern suggests that TNF-α was more closely associated than IL-6 with structural tight junction injury in this experimental setting. Previous work provides a biologically plausible basis for this observation. Haque et al. (24). showed that TNF-α can promote intestinal epithelial tight junction dysfunction through signaling pathways involving NF-*κ*B and related barrier-regulatory mechanisms, providing a possible mechanistic context for the association observed in the present study. Clinical observations from domestic studies have also associated elevated serum TNF-α with worse disease severity in infants with NEC (25).However, TNF-α and IL-6 do not operate on the same biological timescale or functional hierarchy (26). TNF-α is an upstream inflammatory mediator closely coupled to hypoxia/LPS-related NEC induction and NF-*κ*B activation, whereas IL-6 more strongly reflects the downstream systemic acute-phase response. Therefore, the apparent advantage of TNF-α in endpoint samples may partly reflect a mechanism-coupling effect rather than true intrinsic biomarker superiority.

The apoptosis data further supported an association between epithelial injury and tight junction disruption. The progressive increase in intestinal epithelial apoptosis paralleled the reduction in ZO-1 and Claudin-3 expression across groups, suggesting that epithelial cell loss and junctional disruption developed in concert during disease progression. Nevertheless, the present study did not include TNF-α neutralization, NF-*κ*B pathway inhibition, genetic manipulation, or other intervention experiments. Therefore, these findings should be interpreted as correlative and hypothesis-generating. They cannot establish that TNF-α directly causes tight junction loss or epithelial apoptosis in this model.

These findings are also relevant when considered in the context of NEC-related biomarkers. In clinical practice, NEC monitoring still relies partly on systemic inflammatory indicators such as CRP and PCT, but these markers are not specific to intestinal injury and may overlap with neonatal sepsis or other inflammatory states (27). Although the serum TNF-α–tight junction injury association observed here has pathophysiological relevance, it is premature to propose TNF-α as a direct clinical blood biomarker for NEC severity (28). From a practical perspective, the exploratory regression analysis suggests that TNF-α may provide severity-related information without requiring a complex multi-marker model. However, this result should be interpreted cautiously because the analysis was based on only nine complete-case samples and may still partly reflect the between-group severity gradient across the Control, NEC, and NEC + LPS groups. Although peripheral cytokine testing is clinically accessible, the interpretation of serum TNF-α in neonates may be affected by sample handling, assay-platform variability, biological timing, and overlap with other inflammatory conditions such as sepsis. Therefore, the present findings should be viewed as an experimental framework for future biomarker evaluation rather than evidence supporting immediate clinical use in NEC severity assessment.

Several limitations of this study should be acknowledged. First, the correlation and regression analyses were limited by the small complete-case sample size. The pooled correlation and regression analyses included nine animals, and the disease-only correlation analysis included six animals. Therefore, the strong pooled correlations and LOOCV results should be interpreted as exploratory internal findings rather than evidence of externally validated predictive performance. Second, all findings were derived from an animal model, so extrapolation to human NEC should be made cautiously. Third, although the study focused on structural injury to the intestinal barrier, it did not directly determine whether functional permeability impairment occurred in parallel with the observed structural changes. Fourth, all data were obtained at the experimental endpoint, preventing assessment of the temporal sequence among serum TNF-α elevation, IL-6 response, and barrier injury progression. Fifth, no intervention experiments were performed, so mechanistic interpretations remain correlative. Finally, the specificity of TNF-α for NEC-related intestinal barrier injury was not evaluated against other neonatal inflammatory conditions such as sepsis, and the present study did not assess the effects of sample stability or assay-platform variability on serum TNF-α measurement.

## 5 Conclusion

This study preliminarily suggests that NEC progression in neonatal rats is accompanied by progressive structural disruption of the intestinal barrier. Among the peripheral inflammatory markers examined, serum TNF-α showed a closer endpoint association with tight junction injury than IL-6 within this experimental framework. These findings are consistent with the possibility that serum TNF-α may be further evaluated as a candidate peripheral indicator of NEC-associated intestinal barrier injury severity. However, confirmation in larger animal studies, time-course experiments, intervention-based mechanistic studies, and clinical cohorts is required before considering clinical biomarker application.

## Data Availability

The raw data supporting the conclusions of this article will be made available by the authors, without undue reservation.
